# Nationwide trends and geographic variations of diabetes in pregnancy in Thailand: Epidemiology, neonatal outcomes, and healthcare costs under the universal health coverage scheme (2013–2023)

**DOI:** 10.1016/j.pmedr.2026.103452

**Published:** 2026-03-17

**Authors:** Varisara Lapinee, Prapaporn Noparatyaporn, Thipaporn Tharavanij, Petch Rawdaree, Krisada Hanbunjerd, Mahidol University Global Health Research Group, Kwang Mo Yang

**Affiliations:** a ASEAN Institute for Health Development, Mahidol University, Nakhon Pathom, Thailand; b Center for Global Health, Mahidol University, Nakhon Pathom, Thailand; c National Health Security Office, Bangkok, Thailand; d Departmnet of Medicine, Faculty of Medicine, Thammasat University, Pathum Thani, Thailand; e Department of Medicine, Faculty of Medicine, Vajira Hospital, Navamindradhiraj University, Bangkok, Thailand; f Department of Disease Control, Royal Thai Government, Ministry of Public Health, Bangkok, Thailand.

**Keywords:** Gestational diabetes mellitus, Pre-existing diabetes mellitus, Neonatal outcomes, Health expenditure, Thailand

## Abstract

**Objective:**

Diabetes in pregnancy is increasing globally, yet national evidence from Thailand on trends, geographic variation, neonatal outcomes, and healthcare costs remains limited. This study examined temporal trends, regional variation, neonatal complications, and economic burden among beneficiaries of Thailand's Universal Coverage scheme.

**Methods:**

A nationwide retrospective study was conducted using the National Health Security Office electronic claims database in Thailand from 2013 to 2023. The analysis included 103,536 pregnancies affected by diabetes and 14,051 linked infant records. Temporal trends, provincial geographic variation, neonatal outcomes, and direct medical expenditures were examined.

**Results:**

Diabetes in pregnancy increased substantially over the study period. Gestational diabetes mellitus remained predominant, while type 1, type 2, and unspecified diabetes increased after 2020. Healthcare costs rose in parallel. Geographic patterns shifted from minimal provincial differences during 2013–2020 to regional hotspots in central, northeastern, and southern Thailand by 2022–2023. Among infants with available claims records, 87.6% had no complications; neonatal jaundice (9.5%) and congenital malformations (2.3%) were the most common outcomes.

**Conclusions:**

Diabetes in pregnancy in Thailand has increased rapidly with emerging regional concentration and rising healthcare costs. Strengthened surveillance, improved data linkage, standardized diagnosis, and targeted regional prevention strategies are needed.

## Highlights

• Diabetes in pregnancy increased sharply in Thailand from 2013 to 2023.
• Geographic hotspots emerged after 2020 across several provinces.
• Neonatal complications were uncommon but clinically important.
• Healthcare expenditure increased substantially during the study period.

## Graphical abstract

**Figure:**
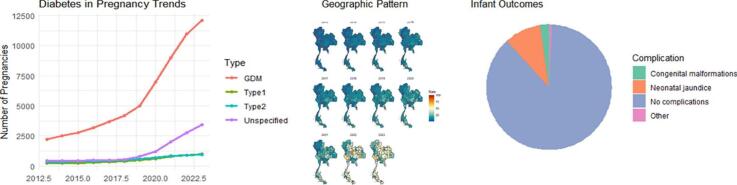

## 1 Introduction

Diabetes in pregnancy has become an increasingly important public health concern worldwide (Nakshine and Jogdand, 2023). Globally, over 16.7% of infants are born from mothers with some form of diabetes (dos Silva Santos et al., 2023; Suda-Całus et al., 2024). Some short-term complications include hypertensive disorders and delivery complications for the mothers, and macrosomia, hypoglycemia, respiratory distress syndrome for the infants (Murray and Reynolds, 2020). For long-term impacts, women with history of gestational diabetes mellitus (GDM) can develop type 2 diabetes and cardiovascular diseases, while the infants born to mothers with GDM can develop obesity, type 2 diabetes, cardiovascular diseases, and neurodevelopmental problems (Song et al., 2018). These burdens increase the demand for healthcare services and medical resources, impacting individuals, families, and healthcare systems (Nakshine and Jogdand, 2023; Song et al., 2018; Staynova et al., 2022).

In Thailand, the prevalence of GDM and pre-gestational diabetes has significantly grown, with rates as high as 22–24% in certain locations due to increasing maternal age, higher pre-pregnancy BMI, and urbanization. (Staynova et al., 2022). However, most available data rely on single hospitals studies or short observation periods, which limits the ability to track long-term shifts, geographic differences, and system-wide impacts. Understanding these patterns is increasingly important for national planning because diabetes in pregnancy influences both immediate obstetric care and long-term health expenditure (Jatavan et al., 2023). In addition, this surge in diabetes during pregnancy is closely linked to adverse maternal and neonatal outcomes, including increased rates of congenital malformations, neonatal jaundice, higher birth weights, and long-term metabolic complications for the offspring (Choudhury and Devi, 2021). Despite the magnitude and rapid progression of the problem, many diabetes affected pregnancies are not linked to comprehensive infant outcome records, and true complication rates are underestimated.

The Universal Health Coverage (UHC) scheme provides health insurance to the majority of Thailand's population, and its administrative claims database offers a unique opportunity to examine national trends (Damrongplasit and Melnick, 2024; Rajatanavin et al., 2022). However, these data have not been fully leveraged to assess how the burden of diabetes in pregnancy has changed over time, how it varies across provinces, and how it affects infants born to mothers with diabetes. In addition, the financial implications for the health system remain poorly understood.

This study used eleven years of electronic claims (e-Claim) data to describe temporal trends, geographic patterns, neonatal outcomes, and medical expenditures related to diabetes in pregnancy among UHC beneficiaries from 2013 to 2023. The findings provide an updated national assessment that can support targeted planning, improve risk-based service delivery, and guide future strategies to reduce maternal and infant morbidity. By integrating epidemiological, clinical, and economic data, this analysis seeks to inform targeted policy, equitable resource allocation, and development of robust national surveillance for maternal and infant health in the face of the ongoing diabetes epidemic.

## 2 Methods

### 2.1 Study design and population

This study utilized secondary administrative claims data from the e-Claim database managed by the National Health Security Office (NHSO) of Thailand. The e-Claim system collects comprehensive medical service utilization records from healthcare facilities nationwide that are registered with the NHSO, covering Thai citizens under the Universal Health Coverage scheme. Data were extracted for the period 2013 to 2023 encompassing 11 years of healthcare service claims related to diabetes in pregnancy.

The target population comprised pregnant women diagnosed with pre-existing diabetes mellitus and gestational diabetes mellitus (GDM), as well as infants born to these mothers. Mother and infant records were identified using International Classification of Diseases, Tenth Revision (ICD-10) diagnostic codes related to diabetes in pregnancy and its neonatal syndromes. The codes used to define mothers with diabetes included O24.0 (pre-existing type 1 diabetes mellitus in pregnancy), O24.1 (pre-existing type 2 diabetes mellitus in pregnancy), O24.3 (unspecified pre-existing diabetes mellitus in pregnancy), O24.4 (gestational diabetes mellitus), and O24.9 (unspecified diabetes mellitus in pregnancy). Infant records were identified using P70.0 (syndrome of infant of mother with gestational diabetes) and P70.1 (syndrome of infant of mothers with diabetes).

A pregnancy episode was defined as a discrete event separated by at least 12 months from any prior birth, allowing identification of distinct pregnancies within the same mother. After restructuring the visit-based dataset into incident pregnancy episodes, the final maternal dataset included 103,536 unique pregnancies affected by diabetes. For infants, each birth was treated as a single independent event, resulting in 14,051 infant records linked to mothers diagnosed with diabetes.

Mother–infant linkage was incomplete in the administrative database. Infant outcome data therefore represent only births with available infant claims records and do not capture all infants born to mothers with diabetes. Consequently, neonatal outcome analyses were interpreted descriptively and should not be considered representative of all diabetes-affected pregnancies.

This study received ethical approval by the Mahidol University Ethical Review Board (Protocol Ref No MU-CIRB 2025/307.1106), approved on 15 July 2025.

### 2.2 Measures

Maternal diabetes status during pregnancy was classified using ICD-10 diagnostic codes recorded in reimbursement claims. Diabetes categories included type 1 diabetes mellitus, type 2 diabetes mellitus, gestational diabetes mellitus, and unspecified diabetes mellitus in pregnancy.

Neonatal outcomes were identified using ICD-10 diagnostic codes recorded in infant reimbursement claims. These diagnoses reflect clinician-reported conditions for administrative purposes rather than standardized research definitions. Neonatal outcomes assessed included neonatal jaundice, congenital malformations, iron deficiency anemia, disorders of carbohydrate metabolism, perinatal hematologic disorders, high birth weight, fetal blood loss. High birth weight was identified using ICD-10 codes indicating excessive fetal growth or large-for-gestational-age status. Congenital malformations included all recorded structural anomalies without differentiation between major and minor defects. Neonatal jaundice reflected clinician-diagnosed hyperbilirubinemia as recorded in claims.

A parallel dataset containing reimbursement transfer records was used to examine healthcare expenditures related to pregnancies complicated by diabetes and their infants. These data represented claims submitted by healthcare providers to the National Health Security Office and included 68,158 reimbursement records associated with maternal diabetes care.

### 2.3 Statistical analysis

Four main analyses were conducted as follows. First, temporal trends in diabetes mellitus (DM) during pregnancy were examined over time, including types of DM cases and the distribution of maternal age. Second, geographical patterns of DM in pregnancy across Thai provinces were identified using K-medoids clustering based on DM rates per 1000 live births. Third, the share of complications among infants born to mothers with DM was assessed. Fourth, a detailed breakdown of total medical expenditures related to DM pregnancies was analyzed by DM type and cost categories. These analyses used incident-based data cleaned and formatted from NHSO visit-based records, focusing on unique pregnancy episodes and births to ensure accurate longitudinal and cross-sectional assessments. Data visualization and descriptive summaries were prepared using Microsoft Excel (Microsoft Office 2019, Microsoft Corporation, Redmond, WA, USA). Distribution maps for Thailand were generated using RStudio running R version 4.4.2.

## 3 Results

**Fig. 1:**
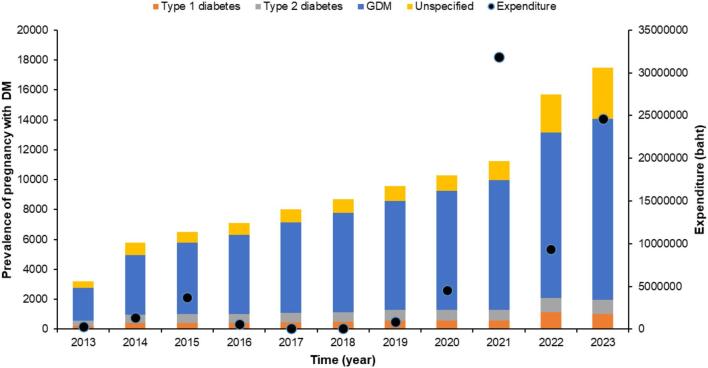
Annual trends in diabetes in pregnancy cases and total healthcare expenditure among pregnant women in Thailand under the Universal Health Coverage scheme, 2013–2023.

The number of pregnancies affected by diabetes rose sharply over the decade (Fig. 1). Gestational diabetes mellitus (GDM) cases increased more than fivefold, from 2209 in 2013 to 12,076 in 2023. Type 1 diabetes rose from 228 to 1015 cases, and type 2 diabetes from 327 to 963 cases, marking a clear expansion in the absolute burden of pre-existing diabetes in pregnancy. Unspecified diabetes also climbed steeply, from 442 cases to 3448 cases, with the most rapid growth occurring after 2020. Together, these trends point to a substantial rise in pregnancies requiring intensified antenatal monitoring, pharmacologic management, and neonatal care. They also suggest increasing pressure on healthcare expenditure within the Universal Health Coverage (UHC) scheme.

Over the study period, total UHC expenditure for diabetes in pregnancy rose from 304,912 baht in 2013 to a peak of 31,873,174 baht in 2021, representing an increase of more than 100-fold. Although spending declined after this peak, expenditure in 2023 remained 24,625,700 Thai baht which is around 80 times higher than in 2013, indicating a sustained and substantial escalation in costs. This pronounced increase in expenditure paralleled the several-fold rise in pregnancies complicated by GDM and pre-existing diabetes.

**Fig. 2:**
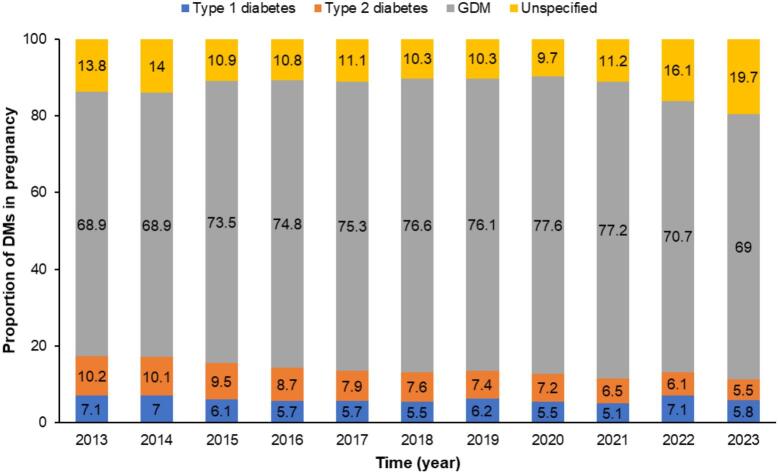
Annual proportion of pregnancies affected by type of diabetes among pregnant women in Thailand, 2013–2023. Categories include type 1 diabetes mellitus, type 2 diabetes mellitus, gestational diabetes mellitus (GDM), and unspecified diabetes recorded in the Universal Health Coverage administrative claims database.

Expressed as proportions, GDM accounted for most diabetes in pregnancy throughout the study period, ranging from 68.9% in 2013–2014 to a peak of 77.6% in 2020, followed by a decline to 69.0% in 2023 (Fig. 2). Type 1 diabetes consistently represented about 5 to 7% of cases. Type 2 diabetes fell from roughly 10% to 5–6%, so pre-existing diabetes combined never exceeded one quarter of all cases. In contrast, unspecified diabetes declined to below 10% in 2020 but then rose sharply to 16.1% in 2022 and 19.7% in 2023, mirroring the apparent proportional decline in GDM. This expansion of the unspecified category suggests increasing misclassification or incomplete coding, especially after 2020, which can dilute apparent GDM trends and obscure the true share of pre-gestational diabetes; such uncertainty complicates precise costing and planning of targeted interventions.

**Fig. 3:**
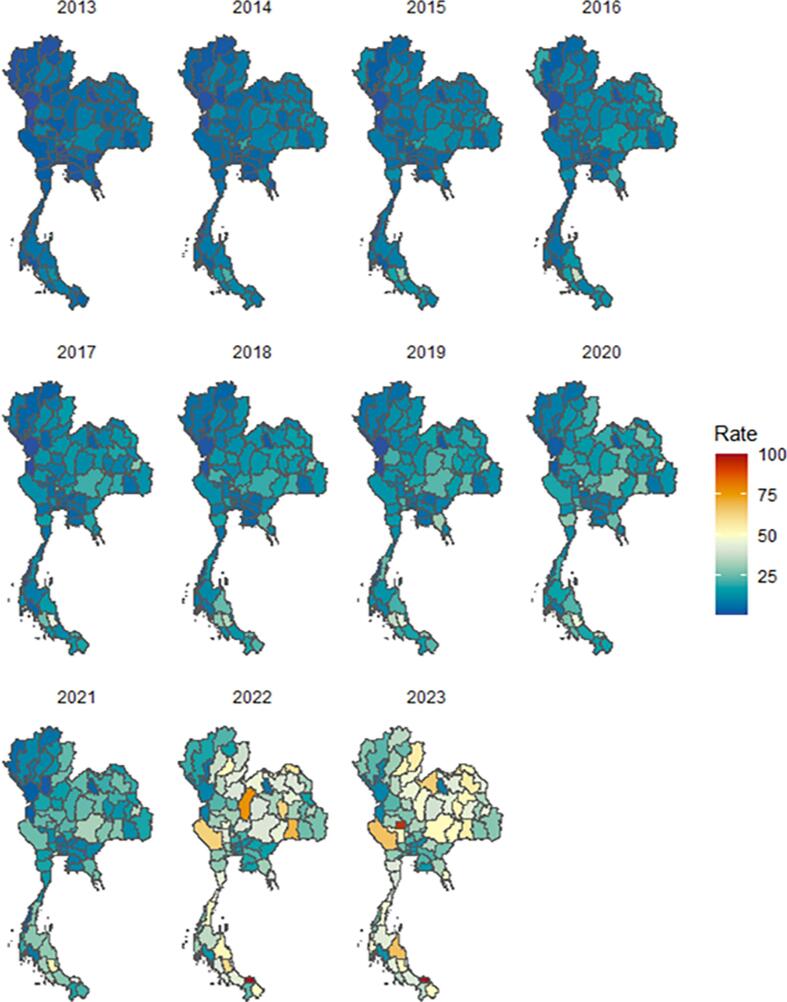
Geographic distribution of diabetes in pregnancy rates per 1000 live births across provinces in Thailand, 2013–2023.

Provincial rates of diabetes in pregnancy remained low and closely clustered from 2013 through 2020 (Fig. 3). During these years, geographic variation was minimal. Although a gradual rise was apparent across most provinces beginning around 2017, the national pattern continued to show a largely uniform burden. A clear shift emerged in 2021. Provincial rates spread into higher ranges, and the visual contrast between provinces increased. By 2022 and 2023, multiple provinces in the central, northeastern, and southern regions displayed markedly elevated rates, forming distinct geographic hotspots. The 2023 map shows the greatest dispersion in rates across the entire study period, with several provinces reaching the highest color categories.

**Fig. 4:**
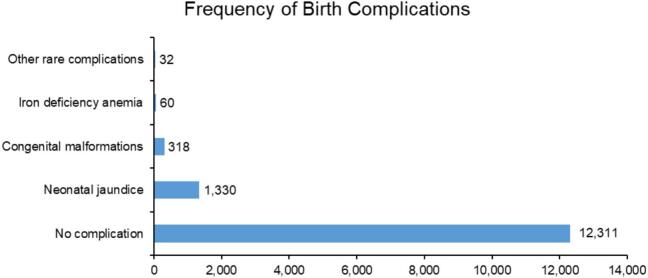
Distribution of neonatal complications among infants born to mothers with diabetes in pregnancy in Thailand, 2013–2023.

A total of 14,051 infants were born to mothers with diabetes during the study period (Fig. 4). Among infants with available claims records, the majority (87.62%, *n* = 12,311) had no documented complications. Among those with adverse outcomes, neonatal jaundice was the most frequently recorded condition, affecting 9.47% (*n* = 1330). Congenital malformations were identified in 2.26% of infants (*n* = 318), making them the second most common complication. Other neonatal outcomes occurred infrequently. Iron deficiency anemia was reported in 0.43% (*n* = 60) of infants, while carbohydrate metabolism disorders and perinatal hematologic disorders were recorded in 0.09% (n = 13) and 0.06% (*n* = 9), respectively. High birth weight appeared in 0.06% (n = 9) of infants, and fetal blood loss was rare, documented in only one infant (0.01%). Although complications were concentrated in a small proportion of births overall, the absolute number of affected infants indicate that adverse neonatal outcomes remain present across a broad clinical spectrum, from common transient conditions such as jaundice to less frequent but clinically significant disorders and structural anomalies.

## 4 Discussion

This study provides a comprehensive national assessment of diabetes in pregnancy within Thailand's Universal Health Coverage scheme over an 11-year period. The results show a substantial and sustained rise in all categories of diabetes in pregnancy, with gestational diabetes mellitus (GDM) driving most of the growth. Although type 1 and type 2 diabetes remained a smaller proportion of cases, their absolute numbers increased, indicating a growing population of women who enter pregnancy with established metabolic disease. Furthermore, the expansion of the unspecified category after 2020 suggests variation in diagnostic or coding practices that may mask the true distribution of gestational and pre-gestational diabetes (Keating et al., 2022).

The increase in diabetes in pregnancy has direct implications for service delivery and health expenditure (Dinh et al., 2024). Higher cases generate greater demand for glucose testing, antenatal visits, pharmacologic treatment, and specialist-led care (Staynova et al., 2022; Dinh et al., 2024). Pregnancies affected by type 1 or type 2 diabetes often require more intensive monitoring, carry higher risks of maternal complications, and lead to increased neonatal admissions (Wyckoff et al., 2025; Vargas et al., 2010; Clement et al., 2025). As a result, even modest increases in the number of women with pre-existing diabetes can contribute disproportionately to total costs (Son et al., 2015). In addition, Thailand has strengthened community- based screening for non-communicable diseases (NCD) through primary care networks, subdistrict health centers, and community health volunteers, with routine screening programs targeting adults at risk of diabetes and hypertension (Rajatanavin et al., 2022). Expanded screening under the UHC system may increase detection of previously undiagnosed diabetes among women of reproductive age, raising recorded prevalence while enabling earlier clinical management.

The geographic analysis reveals an important shift in the epidemiology of diabetes in pregnancy. From 2013 to 2020, provincial rates remained tightly clustered, suggesting relatively uniform risk across the country. After 2020, the distribution widened and clear hotspots appeared in central, northeastern, and southern provinces. This pattern suggests that the drivers of diabetes in pregnancy are intensifying more rapidly in certain areas (Venkatesh et al., 2024). Differences in urbanization, socioeconomic conditions, lifestyle behaviors, and access to screening may contribute to these disparities (Dickens, 2025). The divergence after 2020 may also reflect changes in daily activity, weight gain, and health service use during the COVID-19 era, although further work is needed to clarify these relationships.

The emergence of diabetes in pregnancy “hotspots” has practical implications for maternal and neonatal care. It indicates that a uniform national strategy may not be sufficient and that targeted interventions are likely to be more efficient. Provinces with steep increases may benefit from strengthened screening programs, earlier risk assessment, and focused health promotion initiatives. Thus, incorporating spatial monitoring into routine surveillance can help detect rapid changes in disease burden and guide more responsive planning (Matthews et al., 2019; Takele et al., 2025).

Infant outcomes provide additional insight into the clinical impact of rising maternal diabetes. Although severe outcomes such as metabolic or hematological disorders were rare, their presence across the dataset emphasizes that even uncommon complications carry important implications for neonatal care (Mitanchez et al., 2015; Al Ghadeer et al., 2024). Given the rapid rise in maternal diabetes, the absolute number of affected infants is expected to grow, even if relative proportions remain unchanged. This means stronger preconception care, improved glucose management during pregnancy, and consistent newborn monitoring to reduce preventable morbidity are needed (Wyckoff et al., 2025). The number of infants with available claims records was substantially lower than the number of diabetes-affected pregnancies, indicating limitations in maternal–infant linkage within administrative data systems rather than true absence of births. Although most infants with available records had no documented complications, neonatal outcomes were substantially under-ascertained due to incomplete linkage between maternal and infant claims. Consequently, these findings should be interpreted as descriptive indicators of recorded complications rather than estimates of true population-level risk. However, it is clear that, there is a significant need to implement standardized protocols to effectively keep track of the medical records of these infants.

Taken together, these findings suggest that Thailand is moving from a stable and geographically uniform burden of diabetes in pregnancy toward a more complex pattern characterized by rapid growth, increasing variation between provinces, and measurable neonatal impact. Improving data quality, especially regarding diagnostic coding, will strengthen the accuracy of burden estimates. Harmonizing screening and management practice across provinces may reduce geographic disparities. Strengthening provincial-level surveillance and implementing targeted interventions in emerging hotspots may help slow the acceleration observed after 2020. Continued monitoring of maternal and neonatal outcomes will be important to ensure that health systems remain responsive to the changing epidemiology of diabetes in pregnancy.

Several limitations should be considered when interpreting these findings. First, diagnoses of diabetes in pregnancy reflect recorded clinical coding rather than independently validated case ascertainment, and observed temporal increases may partly reflect improvements in diagnostic coding, documentation completeness, or claims submission practices over time rather than true changes in disease incidence. Second, information on screening coverage, screening methods, and diagnostic thresholds for gestational diabetes mellitus was unavailable, limiting interpretation of temporal trends. Third, neonatal outcomes were substantially under-ascertained due to incomplete linkage between maternal and infant claims, and reported complication proportions should be interpreted as descriptive rather than representative of all births to mothers with diabetes. Finally, the absence of a non-diabetic comparison group precluded estimation of relative risks for adverse outcomes. Despite these limitations, the dataset provides valuable national-level insight into longitudinal trends, geographic variation, and health-system burden of diabetes in pregnancy in Thailand.

## 5 Conclusions

Health Coverage scheme increasing substantially, reflecting growing demand for antenatal monitoring, treatment, and neonatal care. Although most infants with available claims records had no documented complications, neonatal outcomes were incompletely captured because of limitations in maternal–infant linkage within administrative data systems, and reported outcomes should therefore be interpreted as descriptive indicators rather than population-level risk estimates. Together, these findings underscore the need to strengthen national surveillance of diabetes in pregnancy, improve diagnostic coding and maternal–infant data linkage, and prioritize prevention and early risk reduction in high-burden areas through consistent screening, standardized diagnostic practices, and strengthened maternal–infant monitoring systems.

## Declaration of generative AI and AI-assisted technologies in the manuscript preparation process

During the preparation of this work, the author(s) used ChatGPT (OpenAI) in order to improve the grammatical errors and writing flow of the manuscript. After using this tool/service, the author(s) reviewed and edited the content as needed and take(s) full responsibility for the content of the published article.

## CRediT authorship contribution statement

**Varisara Lapinee:** Writing – review & editing, Writing – original draft, Formal analysis, Data curation, Conceptualization. **Prapaporn Noparatyaporn:** Writing – review & editing, Writing – original draft, Formal analysis, Data curation, Conceptualization. **Thipaporn Tharavanij:** Writing – review & editing, Writing – original draft, Formal analysis, Data curation, Conceptualization. **Petch Rawdaree:** Writing – review & editing, Writing – original draft, Formal analysis, Data curation, Conceptualization. **Krisada Hanbunjerd:** Writing – review & editing, Writing – original draft, Formal analysis, Data curation, Conceptualization. **Kwang Mo Yang:** Writing – review & editing, Writing – original draft, Formal analysis, Data curation, Conceptualization.

## Funding

This research received no external funding.

## Declaration of competing interest

The authors declare that they have no known competing financial interests or personal relationships that could have appeared to influence the work reported in this paper.

## Data Availability

Data will be made available on request.
